# Gut-brain-immune interactions in neonatal hypoxic–ischemic brain injury

**DOI:** 10.1186/s40348-026-00252-1

**Published:** 2026-07-27

**Authors:** Josephine Herz, Ivo Bendix, Frederick Orywal, Christoph Härtel, Ursula Felderhoff-Müser

**Affiliations:** 1 Department of Paediatrics I, Neonatology & Experimental Perinatal Neurosciences, University Hospital Essen, University Duisburg-Essen, Essen, Germany; 2 Center for Translational Neuro- and Behavioral Sciences (C-TNBS), University Hospital Essen, University Duisburg-Essen, Essen, Germany; 3 Department of Paediatrics, University Hospital Würzburg, University of Würzburg, Würzburg, Germany; 4 German Center of Infection Research, Partner Site Hamburg-Lübeck-Borstel-Riems, Lübeck, Germany; 5 Interdisciplinary Center of Clinical Research, University of Würzburg, Würzburg, Germany

**Keywords:** Neonatal hypoxic-ischemic encephalopathy, Hypoxia–ischemia, Microbiome, Peripheral immune cells, Gut-brain axis, Gut-immune axis

## Abstract

**Background:**

Neonatal hypoxia–ischemia (HI) is the leading cause of childhood mortality and neurodevelopmental disability. Despite therapeutic hypothermia as the only clinically established treatment to date, outcomes remain poor for a significant proportion of affected infants. Mechanistic understanding has been brain-oriented in the past, however the gut and immune system are increasingly recognized as active modulators of brain injury, recovery and neurodevelopment.

**Main Body:**

Gut microbiota regulate microglial maturation, myelination and blood–brain barrier integrity. Neonatal HI induces gut dysbiosis and barrier failure, associated with neuroinflammation. First, faecal microbiota transplantation experiments in animal models suggest a causal relationship. Clinical data corroborate microbial perturbations in HIE infants, though antibiotic exposure and the NICU environment represent potential confounders. At the level of the immune system, dysregulated peripheral innate and adaptive immune responses are well documented in HI-affected neonates, with some alterations persisting into school age. The microbiota dimension of these immune responses remains largely unexplored, despite well-characterized microbiota–immune interactions in models of adult stroke. Therapeutic candidates include probiotics, human milk oligosaccharides and butyrate, each with preliminary preclinical support but no completed clinical trials in HIE.

**Conclusion:**

Reframing neonatal HI as a systemic gut–brain–immune disease opens up new possibilities for adjunctive therapy and biomarker discovery. Progress requires longitudinal multi-omic clinical cohorts, sex-stratified and disease-phase-resolved preclinical analyses and rigorous evaluation of microbiome-targeted interventions.

## Background

Neonatal encephalopathy due to hypoxia–ischaemia (HI) remains a leading cause of neonatal mortality and lifelong neurodevelopmental disability. Despite the implementation of therapeutic hypothermia as standard of care, a substantial proportion of affected infants experience adverse neurological outcomes, underscoring the need for adjunctive therapies. Mechanistic insight into HI pathophysiology has historically been brain-oriented, but emerging evidence indicates that early-life brain injury induces systemic responses in the gut and the immune system, which together shape secondary injury and recovery [1, 2].

The gut–brain–immune axis describes bidirectional communications between these three organ systems. In neonates, this network is uniquely dynamic and vulnerable, as microbial colonization of the gut, postnatal maturation of the immune system, and ongoing brain development occur simultaneously and influence each other. Neonatal HI can disrupt intestinal integrity and barrier function, facilitating translocation of microbial products that may amplify neuroinflammation through, for example, microglial activation [3]. Beyond microglia, microbiota and their metabolites shape intestinal and peripheral immune cell development [4, 5]. Furthermore, microbiota-mediated alterations of T cells and neutrophils are increasingly recognised as modulators of ischemic brain injury in adults [6, 7]. Far less is known about these interactions in the neonatal context.

Reframing neonatal HI as a systemic gut–brain-immune disease, rather than a purely cerebral one, could open up new possibilities for biomarker discovery and therapeutic interventions, including microbiome modulation, restoration of intestinal barrier integrity, and targeted immunomodulation. In this review, we summarise current preclinical and clinical evidence on the role of gut–brain-immune interactions in neonatal HI brain injury. We discuss mechanistic links between gut microbial dysbiosis, peripheral immunity and neuroinflammation, highlight methodological challenges of current research, and outline translational strategies aimed at improving neurodevelopmental outcomes.

## Interactions between gut microbiota, central nervous system and immune system development

### Microbiota and brain development

Gut microbiota actively participate in central neurodevelopmental processes including neurogenesis, myelination, blood–brain barrier formation and microglial maturation [8, 9]. Comparison of germ-free (GF) and conventionally colonized mice has shown that maternal and early-life microbial exposures shape microglial development, contributing to autism spectrum disorder (ASD)-like behaviours that can be partially reversed through postnatal microbial or metabolite supplementation [5, 10]. A healthy microbiome is also required for proper cortical myelination at critical windows of neurodevelopment [11]. Mechanistically, the short-chain fatty acid (SCFA) butyrate promotes remyelination, whereas the microbial metabolite 4-ethylphenyl sulfate (4EPS) impairs oligodendrocyte maturation and disrupts myelination in limbic regions, contributing to anxiety-like behaviour [12, 13]. Many of these studies, however, rely on terminal phenotyping in fully GF mice or on treatment beyond the early postnatal window, leaving the precise developmental windows and the specific microbial taxa shaping brain development insufficiently defined. A recent study in extremely preterm infants showed that Klebsiella overgrowth was highly predictive for brain damage and associated with a pro-inflammatory state, elevated γδ T cell levels and reduced secretion of neuroprotectants at term equivalent age [14]. Although this kind of studies, providing the link between the triangle microbiome – immune system and CNS are extremely relevant, long-term follow up data will be equally important and causality needs to be proven. A causal link between neonatal microbiota and human brain development has been described recently. Infants with higher gut microbial diversity and evenness showed improved cognitive performance, and faecal microbiota transplantation (FMT) from these infants into GF mice transferred enhanced memory function at, associated with enrichment of *Phocaeicola, Bacteroides* and *Bifidobacterium* [15].

### Microbiota and immune system development

Studies in GF mice and in models of perinatal antibiotic exposure define a critical window of opportunity for immune maturation shortly after birth [16, 17]. Reduced microbial diversity or early-life antibiotic exposure is associated with an increased risk of allergic disease in humans [18]. In mice, microbiota are essential for the development of regulatory T cells and TH17 cells [5, 18]. Early microbial exposure also supports the induction of innate γδ T cells [19], while limiting the accumulation of pro-inflammatory iNKT cells, whose expansion in the absence of neonatal microbial exposure increases susceptibility to colitis in adulthood [20]. On the myeloid side, perinatal antibiotic exposure in mice induces neutropenia and increases susceptibility to sepsis, which can be partially reversed by reconstitution with age-matched microbiota [21]. Together, these observations highlight the fundamental role of the microbiota in shaping balanced immune responses, with lifelong consequences for health and disease [4, 5, 16–18]. At the molecular level, microbiota-derived metabolites, especially SCFAs, regulate immune programming, including epigenetic modifications relevant for T cell differentiation and function [22, 23]. Microbial metabolites also modulate innate immune priming [4, 5, 24, 25], although the underlying mechanisms are context-dependent and many of these data derive from adult studies. The impact of perinatal microbiome metabolites on epigenetic programming of developing immune cells is only beginning to be understood.

The reverse direction, namely how peripheral immune cells shape the microbiome is comparatively understudied. In adult mice, extrathymically generated peripheral Tregs modulate the metabolic function of the intestinal microbiota [26]. In neonates, reduced accumulation of myeloid-derived suppressor cells (MDSCs) coincided with an altered microbiome composition, although a direct causal relationship remains to be established [27].

### Immune contributions to brain development

Physiological CNS development depends on a range of immune signals, including TLR signalling, cytokines and complement proteins, which contribute to neurogenesis and synaptic refinement [5]. The interrelation between immune cells and brain development is best illustrated by microglia [28]. Although the brain has traditionally been viewed as immune-privileged, the discovery and characterization of brain-resident T cells in human and mouse brains is challenging this paradigm [29–31]. T cell subsets, including regulatory T cells in the meningeal space and choroid plexus have been linked to social and cognitive behaviour [32, 33], but many studies rely on adult mice leaving open whether observed brain dysfunctions are of developmental origin. A recent study in Rag2-/- mice demonstrated that adoptive transfer of neonatal lymphocytes could rescue spatial memory deficits [34]. These findings indicate that lymphocytes can mediate neuronal plasticity during brain development, although the specific lymphocyte subtype that mediates these effects remains unclear. Furthermore, a direct link to microbiota maturation in neonates is missing. The clinical relevance of the triangle gut, immune system and brain in neurodevelopment is illustrated by ASD, where gut microbiota and brain-resident CD4 + T cells together shape behavioural outcomes [35].

## Impact of neonatal hypoxia–ischaemia on the microbiome

### Clinical evidence

While several studies suggest a link between the gut microbiota and the immune system, these mainly focused on preterms and rarely included neurological endpoints [14, 36–38]. In the case of term HIE only very few data exist. In a cohort of 28 newborns with HIE undergoing therapeutic hypothermia (HT), stool samples obtained at a median age of 68 h with follow-up at 151 h in 20 of these infants were compared with single stool samples from 19 healthy newborns [39]. The HIE/HT group showed lower microbial diversity and a higher proportion of staphylococci [39]. A two-year follow-up of 9 HIE and 8 control infants reported no significant impact of HIE/HT on overall gut microbial composition or metabolic profile, although alpha diversity and *Bacteroides* abundance were reduced in the HIE/HT group [40]. Both studies share limitations typical of clinical HIE microbiome research: small sample size, group differences in antibiotic exposure (all HIE/HT infants versus essentially none of the controls), unmatched delivery mode, NICU admission, mother–infant separation and feeding regimen. Together, the limited clinical evidence points to microbiome alterations in HIE/HT infants, but the relative contributions of HI injury per se, antibiotics and other confounding factors such as NICU environment and feeding practices cannot yet be resolved. Robust, well-controlled longitudinal cohorts with appropriate stratification and standardized neurodevelopmental endpoints are urgently needed.

### Experimental evidence

The relationship between maternal microbial perturbation, maternal inflammation and neurodevelopmental disorders is comparatively well established [8]. For postnatal noxious events such as HI, the evidence is more fragmented, but recent research has uncovered first mechanistic insights. In a mouse HIE model incorporating antibiotic treatment from embryonic day 15 and selective postnatal recolonization with either pathogenic *E. coli* or commensal *Bifidobacterium infantis* from postnatal day 5 (P5) revealed dampened HI-induced cytokine alterations for the latter, whereas pathogenic recolonization amplified them [41]. Nevertheless, overall brain edema was unaffected [41]. A potential limitation acknowledged in the study includes antibiotic delivery by maternal drinking water, hampering conclusions about intestinal and systemic exposure of pups to antibiotics. Furthermore, a causal relationship was not established. A causal link between HI-induced microbiome changes and neuroinflammation has more recently been provided by faecal microbiota transplantation (FMT) experiments [42]. Increased abundance of *Proteobacteria*, specifically *Enterobacteriaceae* and *Prevotella* in HIE rats 3 days after the insult were paralleled by reduced tight-junction protein expression in gut tissues, increased circulating LPS and enhanced neuroinflammatory responses in the hippocampus [42]. FMT from sham animals into HI recipients ameliorated these changes, while FMT from HI animals into sham recipients was sufficient to induce neuroinflammation [42]. Oral dexamethasone (DEX) administration from postnatal day 7 reduced systemic and colonic cytokines and improved long-term outcomes [42]. Open questions relate to the efficacy of FMT engraftment and considering the reported potential neurotoxic effects of DEX on the neonatal brain [43], further validation will be important before clinical translation. A complementary study extended these observations to the underlying molecular pathways [3]. In a neonatal rat HIE model the gut microbiota was again enriched in *Proteobacteria* with overgrowth of *Enterobacteriaceae*, accompanied by gut barrier leakage and elevated circulating LPS three days after the insult [3]. Inflammatory signaling pathways were enriched in both colon and hippocampus, mechanistically linking microbiome dysbiosis to TLR4/MyD88/NF-κB-driven neuroinflammation [3]. Nevertheless, similar to the aforementioned studies the observational period for microbial changes following HI was restricted to the first 72 h. Whether the microbial community resolves toward a sham-like state, stabilizes in a perturbed configuration or transitions through a distinct subacute phenotype remains unknown. These temporal changes might be of particular relevance for brain pathophysiological processes but also for peripheral immune cell functions. For instance, brain infiltrating neutrophils display disease stage dependent phenotypic and functional dichotomy at acute and subacute disease stages [44]. Aligning microbiome characterization with evolving and resolving neuroinflammatory responses will need to be investigated in future studies.

Beyond the microbial community itself, HI alters gut structure and intestinal immunity, including increased pro-inflammatory cytokine expression and reduced tight junction protein expression in epithelial cells [3, 42]. Furthermore, in a preterm ovine global HI model, gut T cell numbers and neutrophils were elevated and smooth muscle thickness was increased, alterations that may compromise contractility [45]. The contribution of microbiome-shaped peripheral immune cells to brain injury is well characterized in adult stroke [6, 7, 46], but has not been investigated in neonatal HI.

## Brain–immune interactions in neonatal HI — and their links to the microbiota

### Microglia

Microglia are the brain’s tissue-resident macrophages and the principal cellular drivers of secondary inflammatory injury after neonatal HI. Their role, however, is more complex than a simple effector–injury relationship [47]. Microglia simultaneously regulate physiological neurodevelopmental processes (synaptic pruning, oligodendrocyte support, vascular patterning) and respond to injury, with pro-inflammatory activation at critical developmental windows disrupting connectivity and myelination [48]. Conversely, selective depletion of microglia by intracerebral clodronate liposomes prior to transient middle cerebral artery occlusion in P7 rats increased injury volume and cytokine accumulation, demonstrating that microglia also contribute to endogenous defence [49]. Microglia are therefore not categorically injurious but rather context-dependent contributors whose net effect depends on activation state, timing and developmental stage. This functional plasticity is reflected in the dynamic phenotypic profile of microglia after HI [50, 51]. Pro-inflammatory microglial activation has been mechanistically linked to white matter injury through reduced Wnt/β-catenin signaling, and pharmacological restoration of this pathway with a microglia-targeted Wnt agonist prevented hypomyelination and behavioural deficits in a preclinical model of preterm birth-related brain injury [52]. These findings highlight that the relevant therapeutic target is not microglia presence per se but the molecular regulators that drive activation toward injurious or regenerative phenotypes during specific developmental windows.

Microglia maturation, morphology and innate immune responsiveness depend on host microbiota. GF mice display global microglial defects, partially rescued by microbial recolonization, SCFA receptor FFAR2 mediates SCFA-driven microglial homeostasis under steady-state conditions [10]. In neonatal HI, *Proteobacteria* overgrowth and gut barrier failure were correlated with microglia activation in neonatal rats and FMT from HI animals into antibiotic-treated recipients reproduced microglia activation and cognitive deficits [3, 42]. These findings provide causal evidence that the dysbiotic neonatal microbiome in rodent HIE models is sufficient to drive microglial pathology after HI. Whether this also applies to human HIE and whether, HI-induced microglial activation conversely shapes the developing gut microbiome, for instance via vagal efferents, HPA-axis signaling or cytokine leakage through the injured blood brain barrier remains to be explored.

### Peripheral immune cells

Neonatal immunity is tightly programmed rather than deficient, balancing host defence with the prevention of excessive inflammation in a microbially naïve organism. The capacity of neonatal immune cells to mount robust effector responses is evident in sterile inflammation caused by HI [1]. How the neonatal microbiome shapes these populations during a window of simultaneous immune and microbial maturation and how HI might disturb this co-evolution is largely unexplored. The following sections discuss different immune cell populations focusing on what is established for HI brain injury and what can be inferred regarding a potential bidirectional interaction with the gut microbiome.

Pre-clinical and clinical data demonstrate peripheral neutrophilia within the first hours after the insult [53, 54], associated with poor neurodevelopmental outcome in term infants with neonatal encephalopathy (NE) [53]. Neutrophils from NE infants display a dysregulated, pre-activated profile and elevated neutrophil-associated inflammatory cytokines (e.g., IL-8) were detected in serum and CSF of moderate-to-severe NE infants [55, 56]. The pre-activated profile of neutrophils from NE infants is supported by increased expression of CD11b and TLR-4, the latter associated with mortality [57]. Neutrophil function within the injured brain itself remains difficult to assess in patients. Recent preclinical work in a mouse model showed a biphasic neutrophil infiltration pattern with peaks at one and seven days post-HI [44]. These two waves are functionally dichotomous, with early infiltrating neutrophils displaying a hyperactivated, tissue-damaging phenotype, whereas late infiltrating neutrophils exhibit an angiogenic phenotype supporting vascular and oligodendrocyte regeneration [44]. Acute neutrophil depletion was neuroprotective, while delayed depletion impaired regeneration and worsened myelination and behavioural outcome at young adult age [44], supporting that, similar to microglia, the therapeutic target is not neutrophils per se but the specific phenotype at a given disease phase.

The microbiota–neutrophil link is well documented in adult stroke where neutrophil phenotype and function is modulated by the microbiota [7]. Concerning neonatal neutrophils, findings are limited to infectious contexts. Perinatal antibiotic exposure in mice induces neutropenia and increases sepsis susceptibility, both reversed by reconstitution with age-matched microbiota [21], demonstrating that the neonatal microbiome targets the neutrophil compartment. Whether systemic antibiotic exposure routinely received by NE neonates may shape the neutrophil compartment via modulation of the microbiome remains an open question. Furthermore, whether the pre-activated, TLR-4-high neutrophil phenotype observed in NE infants reflects microbiome-derived priming, e.g. via increased systemic LPS exposure secondary to gut barrier dysfunction [3, 12], displays an additional hypothesis. The biphasic neutrophil dynamics described by Richter et al. add a temporal layer to this question with the acute hyperactivated phase coinciding with the documented window of gut barrier failure and *Proteobacteria*-dominant dysbiosis whereas the regenerative phase falls into a window for which the gut microbiome remains to be characterized. Whether HI-induced neutrophilia [53, 54] and increased neutrophil accumulation in the gut [45] conversely modify the establishing microbial community is also unknown.

Invariant NKT (iNKT) cells and Vδ2 γδ T cells are expanded in neonates with NE, in school-age children post-NE and in children with cerebral palsy compared with age-matched controls [58]. Upon ex vivo stimulation, T cells, NK cells and Vδ2 γδ T cells from NE neonates produced more inflammatory cytokines than control cells, suggesting prior priming [58]. Large numbers of γδ T cells were also detected in post-mortem brain tissues of preterm human infants and in tissues of small and large animal models of perinatal HI [59]. Depletion of these cells provided neuroprotection against HI-induced brain injury in preterm-equivalent rodents [59]. The microbiota link appears particularly relevant for these unconventional lymphocyte populations, as they are among the most microbiota-sensitive subsets. Colonisation of GF animals with a conventional microbiome during the neonatal period, but not in adulthood, protects against mucosal iNKT accumulation and associated colitis pathology, defining a critical window during which microbial exposure constrains the iNKT compartment [20]. It remains to be clarified whether the persistent iNKT expansion documented until school age in NE infants [58] might be related to, or even be caused by, HI-associated perinatal microbial perturbations. Gamma delta T cell development in humans is similarly shaped by early microbial exposure [19] and work in adult mice has shown that gut-resident γδ T17 cells migrate to the injured brain contributing to cerebral ischaemic injury [6]. In addition to the damaged brain, parenchymal T cells in the subfornical organ, important for maintaining brain homeostasis, are primed by the gut microbiome and traffic from gastrointestinal and adipose tissues to the brain, with their numbers being modulated by gut microbial alterations [60]. Beyond the brain parenchyma, the meninges contain a resident γδ T cell population that modulates synaptic plasticity and short-term memory in adult mice via IL-17 production [61]. Furthermore, gut-licensed IFNγ-producing NK cells home to the meninges and shape an immune-regulatory astrocyte phenotype during CNS inflammation [62]. These findings suggest a more general gut–meningeal immune circuit [63] whose ontogeny in the postnatal period and its potential disruption by HI-induced dysbiosis remains to be investigated. Furthermore, whether gut-to-brain T cell trafficking, as described in adults, is established during the neonatal period and a potential impact of neonatal HI has yet to be explored.

Circulating B cell numbers are elevated in neonates and school-age children with NE and cerebral palsy compared with age-matched controls [58]. While the contribution of B cells to neonatal HI brain injury, remains poorly defined [1], the bidirectional B cell–microbiota relationship was recently demonstrated in neonatal rodents by probiotic exposure in the perinatal period with *Lactobacillus acidophilus* and *Bifidobacterium bifidum,* resulting in reduced B cell frequencies in the intestine at weaning [64]. A potential link between gut and brain is provided from work in adult animals, revealing that gut-educated IgA-producing plasma cells home to the dural venous sinuses, where their B-cell receptor repertoire mirrors that of the gut and confers protection against blood-borne pathogens reaching the CNS [63, 65]. Whether this gut–meningeal axis is established during the postnatal window in which the microbiome and the meningeal immune compartment co-mature, and whether HI-induced dysbiosis disrupts its assembly, remain unknown.

**Fig. 1:**
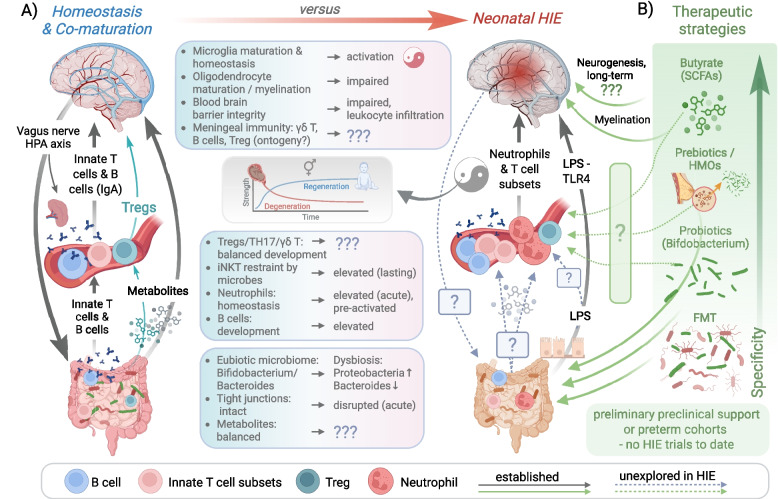
Gut–brain–immune interactions in neonatal hypoxic–ischemic encephalopathy (HIE). **A** Bidirectional communication between gut microbiota, immune system and brain under homeostasis (left) *versus* neonatal HI. During homeostatic co-maturation, microbiota and their metabolites support microglial and oligodendrocyte maturation and development of immune cells, including regulatory T cells (Tregs), innate T cell subsets and IgA-producing B cells. Brain homeostasis depends on B cells and T cells (e.g. Tregs and γδ T cells) in the parenchyma and meninges. Ontogeny of these brain lymphocytes remains unclear. Neonatal HI leads to microglial activation, impaired myelination, blood–brain barrier disruption with leukocyte infiltration. The yin–yang symbols mark context-dependent, dual (injurious vs. reparative) roles of microglia and infiltrating immune cells, whose net effect depends on activation state, timing and developmental stage as well as sex (grey box). Documented peripheral immune alterations include acute neutrophilia with a dysregulated, pre-activated profile, lasting iNKT-cell expansion and elevated B-cell numbers. Frist preliminary studies suggest an HIE-induced gut dysbiosis and acute tight-junction disruption. The resulting increase in circulating LPS may amplify neuroinflammation via two routes: direct modulation of microglia through TLR4, and, so far unexplored, potential priming of peripheral neutrophils secondary to gut barrier dysfunction. Specific pathways and metabolites mediating further alterations in immune cells and brain inflammation as well as communication pathways from the injured brain to the gut remain to be investigated. **B** Microbiome-directed therapeutic strategies, ordered along a spectrum from least to most targeted manipulation: faecal microbiota transplantation (FMT, whole-community transfer, safety issues), defined probiotic strains (e.g., Bifidobacterium), prebiotics/human milk oligosaccharides (HMOs) that select for beneficial taxa, and supplementation with single microbial metabolites (butyrate/short chain fatty acids (SCFAs)). Proposed actions of metabolites include protection from hypomyelination and promotion of neurogenesis. The impact of the suggested therapies on immune cell function and maturation is unclear in neonatal HIE

Combing existing experimental and clinical findings, plausible mechanistic hypotheses emerge (Fig. 1), which are however unproven in human neonatal HIE. As documented in animal models [3, 42, 45], HI results in enrichment of specific bacterial taxa (e.g. *Enterobacteriaceae*) combined with disturbed gut barrier functions, leading to a systemic increase in immune stimulating bacterial compounds (e.g. LPS) that mediate neuroinflammation through TLR4 signalling in microglia, but possibly also through peripheral immune cell priming, which might explain the pre-activated neutrophil and the expanded unconventional T-cell phenotypes documented in NE infants [55–58]. In view of the complex role of peripheral immune cells in HI pathophysiology [1, 44], microbiota-primed leukocyte subsets may by itself contribute to tissue injury or in later disease phases promote recovery. Restoring eubiosis in all disease phases may improve short- and long-term neurodevelopmental outcome. However, to date, several gaps of evidence remain experimentally and clinically. For instance, the synthesis applies mainly to term and near-term infants with moderate-to-severe HIE, treated with therapeutic hypothermia, while the supporting mechanistic data derive from studies in P7 preterm-equivalent rodent models, not treated with hypothermia.

Taken together, across different peripheral immune populations characterized in neonatal HI, the microbial dimension has been largely ignored in this disease setting. The neonatal window of opportunity [4, 5, 16, 17] offers a mechanistic framework for reinterpretation of immune alterations documented in NE cohorts. The inverse direction, namely immune-cell-driven shaping of the neonatal microbiome, is essentially unexplored. Closing these gaps will require appropriate pre-clinical experimental designs that combine defined microbial communities with cell-type- and disease-stage resolved peripheral and brain immunophenotyping, along with longitudinal clinical cohorts that integrate microbiome and immune trajectories. These complex approaches will hopefully help to refine currently discussed therapeutic concepts.

## Targeting the gut–brain–immune axis: therapeutic strategies

Adjunctive neuroprotection beyond therapeutic hypothermia has so far been difficult to be established clinically. The phase III HEAL trial showed that high-dose erythropoietin added to hypothermia did not reduce death or neurodevelopmental impairment, despite achieving target drug exposure [66]. Allopurinol is under phase III evaluation as an adjunct to hypothermia (ALBINO, NCT03162653); the statistical analysis plan has been published and the primary two-year outcomes are awaited [67, 68]. Melatonin remains early-phase and experimental, with the multicentre ACUMEN study designed as a first-in-human phase I safety and dose-escalation trial of intravenous melatonin during hypothermia [69]. None of these trials specifically targeted the gut–brain–immune axis, but instead focused on systemic or direct neuroprotective mechanisms adjunctive to therapeutic hypothermia. Given the limited success, microbiome-directed proposals must overcome significant hurdles to prove their clinical value.

Microbiome-directed strategies for neonatal HI can be ordered along a spectrum from least to most targeted manipulation, beginning with whole-community transfer and ending with the supplementation of single bacterial metabolites. At the broadest end of this spectrum, FMT in rodents was shown to reproduce injury phenotypes in healthy recipients, demonstrating that the dysbiotic community is sufficient to drive neuroinflammation [3]. In future analyses, the specific bacterial taxa that need to be replaced or supplemented will need to be defined, as FMT in extremely vulnerable neonates raises safety concerns that are unlikely to be resolved in the near future. A more targeted alternative is the introduction of defined bacterial strains. Probiotic interventions can ameliorate symptoms in animal models of neurodevelopmental disorders [70] and perinatal exposure to *Lactobacillus acidophilus* and *Bifidobacterium bifidum* profoundly modified immune cell composition in the intestine, liver, lung and thymus [64]. The importance of specific microbiota for proper immune system development was also recently shown in term born infants, revealing that a lack of *Bifidobacteria* and in particular the depletion of genes required for HMO utilization from the metagenome was associated with systemic inflammation and immune dysregulation early in life [36]. This study is complemented by an additional interventional multicenter, double-blinded, placebo-controlled phase III trial including 643 preterm infants, randomized to *Bifidobacterium longum* subsp. *infantis*, *B. animalis* subsp. *lactis* and *Lactobacillus acidophilus* supplementation versus placebo [38]. Even though no neuroprotection endpoint was included, this treatment resulted in rebalancing the microbiome toward the eubiotic profile of healthy term infants at both genus and species level, without adverse safety signals [38]. With regard to neurodevelopmental outcome, data are limited. A recent multiomic study including 60 extremely preterms provided first associations between brain maturation deficits and alterations in microbiota and immune cell responses [14]. Nevertheless, the daily probiotic administration in this cohort did not completely protect from perinatal white matter injury [14]. To date clinical studies in the context of neonatal HIE-induced brain injury are lacking and preclinical evidence for neuroprotection by probiotics in neonatal HI models is rare. In a combined HI + LPS rat model, a multi-strain probiotic attenuated microglial activation and postnatal seizures, restored cortical GABAergic interneurons and elevated faecal and CSF butyrate [71]. In neonatal mice subjected to HI, selective postnatal recolonisation with *B. infantis*, following maternal antibiotic depletion, reduced pro-inflammatory cytokines compared with pathogenic *E. coli* recolonization [41]. In addition, *B. animalis subsp. lactis* pre-treatment in LPS-challenged neonatal rats dampened central and peripheral immune responses with neuroprotective effects [72]. Despite heterogeneity in strains, doses and insult types, these studies provide preliminary indications for neuroprotection by probiotics, but further systematic studies with clear therapeutic designs are needed.

A complementary route to target the microbiome is via the substrates that select for beneficial taxa rather than introducing the taxa themselves. Prebiotics, non-digestible substrates that selectively promote beneficial bacterial growth, have been shown to modify gut microbiota composition and intestinal immune function [73, 74]. Human milk oligosaccharides (HMOs) act as selective prebiotics for *Bifidobacterium* and *Bacteroides* and as direct modulators of mucosal immunity and the developing brain [75]. Mechanistically, it was shown in adult mice that 3’-/6’-sialyllactose prevents stressor-induced anxiety-like behaviour and dentate gyrus neurogenesis defects [76]. Direct evaluation of prebiotics or specifically HMO-mediated neuroprotection in models of term-born neonatal HI is not available, but HMOs have shown protection from white matter injury and improved interneuron development in a model of preterm birth-related brain injury induced by prenatal inflammation combined with postnatal hypoxia [77]. HMOs may be a potential treatment candidate, since they are now produced at scale and approved for use in infant formula, lowering the regulatory threshold for clinical evaluation in NE infants who cannot be exclusively breast-fed, especially during the first three days of life during hypothermia. Nevertheless, no efficacy data in HIE exist and any benefit remains hypothetical at this stage. The feeding regimen during therapeutic hypothermia is itself a primary determinant of microbial assembly. Enteral feeds were frequently withheld or minimised during cooling, fearing an increased risk of complications (e.g. NEC), even though the overall risk of NEC in late-preterm and term infants is very low (< 1%) and first studies show beneficial effects of enteral feeding during hypothermia [78]. Timing of initiation, volume, and feed advancement is highly variable between centres making interpretation of microbiome assessments across different study sites even more difficult. Another issue to consider is the use of human milk versus formula exposure which will differentially shape *Bifidobacterium* colonisation and the availability of HMOs [36, 75]. Feeding practice is therefore a major confounder in clinical HIE microbiome studies, for which any microbiome-directed intervention must account for.

At the most targeted end of the spectrum, supplementation with the bacterial metabolites themselves bypasses the question of which taxa substrates to enrich. Subcutaneous administration of sodium butyrate (SB), a SCFA, from P7 over five days has been shown to increase neurogenesis and oligodendrocyte precursor cell numbers at 14 days post-HI [79], while the delayed rise in pro-inflammatory CXCL10, IL-1β and COX-2 was diminished [80]. A more recent study additionally reported increased expression of the synaptic protein PSD-95 [81]. However, behavioural outcomes in young adulthood were not, or were only minorly affected [79], raising the question of how durable the long-term impact of early-life metabolite supplementation is. In a complementary approach, intragastric SB administration over two weeks reversed acute HI-induced shifts in the *Firmicutes*/*Bacteroidetes* ratio towards sham levels, restored histone 3 lysine 9 crotonylation (H3K9cr) and the expression of neurotrophic genes including *Bdnf, Gdnf, Cdnf* and *Manf* [82]. Nevertheless, behavioural assessment in that study was performed at two weeks of age, i.e. pre-weaning and results are based on a cohort of n = 3. Future work will need to address these limitations with appropriately timed assessments and larger group sizes. Beyond SCFAs, the metabolite space relevant to neonatal HI remains largely unexplored. For instance, tryptophan-derived indoles act on the aryl hydrocarbon receptor to shape microglial and astrocyte phenotypes limiting neuroinflammation [83, 84]. Bile acids signal through FXR and TGR5 at the gut-brain interface, even though the relative contribution of peripheral and CNS-cell derived compounds to their neuromodulatory effects are not fully understood [85]. Whether these physiological axes via metabolites are perturbed after neonatal HI, and whether they can be targeted therapeutically, has not been examined.

## Perspectives

The gut–brain-immune axis in neonatal HI is a young but evolving field positioned to move from descriptive correlation towards mechanistic causality and clinical translation. To summarize the previous sections, main findings are graded by the strength of evidence in Table 1, distinguishing four levels: human association (correlational, typically confounded by clinical care), animal causality (interventional, e.g. faecal microbiota transplantation), extrapolation from adult stroke or non-HI inflammatory models, and therapeutic speculation.

**Table 1:**
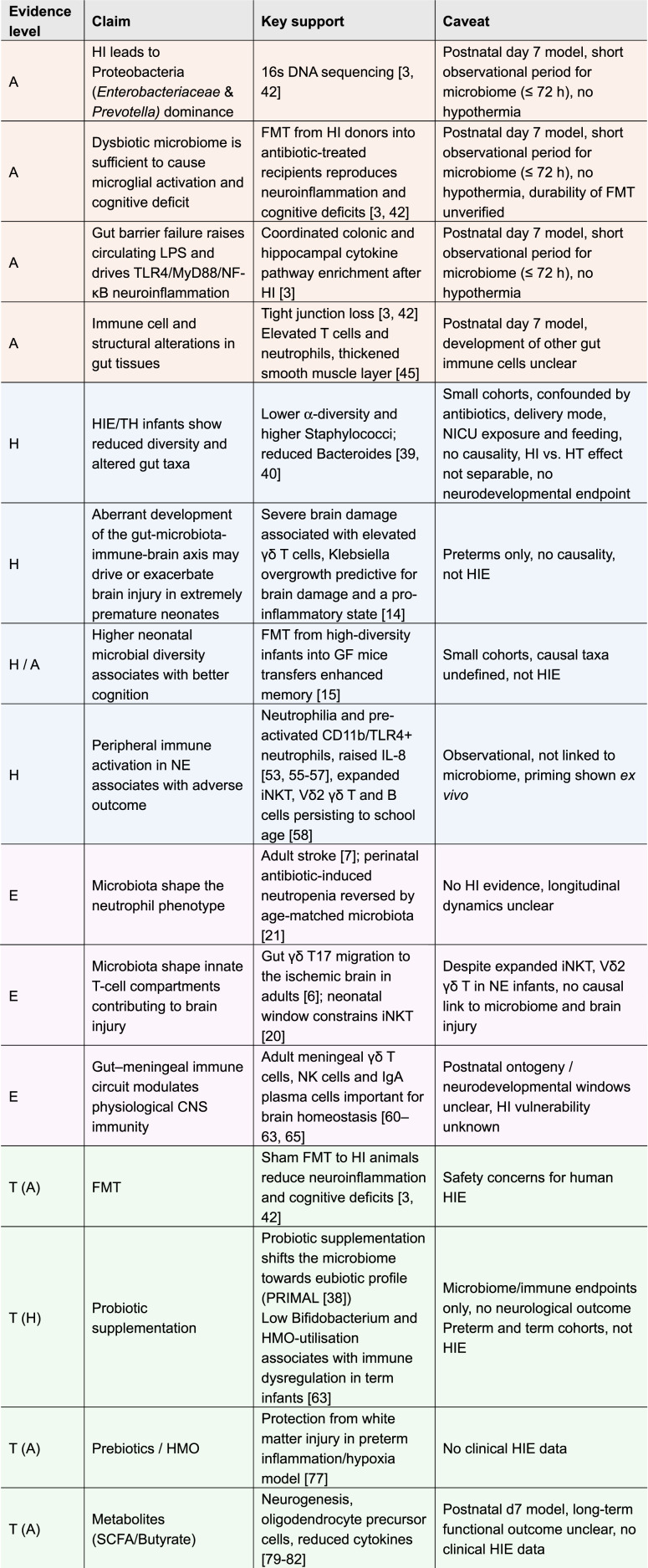
Levels of evidence underlying the principal claims of this review

*A* Animal causality*, H* Human association*, E* Extrapolation from adult stroke or non-HI models*, T* Therapeutic speculation

From this overview, two research priorities can be defined: unaddressed mechanistic links and conceptual gaps in preclinical and clinical study designs (Table 2).

**Table 2:** Research priorities

Mechanistic gaps	Study design requirements
Meningeal-immune-microbiome interaction	Longitudinal multi-omics linked to neuroimaging & outcome
Disease phase resolved mechanisms ('window of opportunity')	Antibiotic-matched control groups & consideration of co-variables: NICU environment and feeding
Causal taxa & metabolite pathways (beyond SCFAs)	Sex as biological variable
Microbiota-shaped immune responses & neurodevelopment (long-term programming)	Interventional microbiome-targeted trials

At the conceptual level, two questions remain. First, sex differences are well documented separately for the developing brain, immune system and gut microbiota [86–88]. HI itself produces sex-specific patterns of lesion size, microglial activation, cell-death pathways and peripheral immune cell function [47, 89–92]. Whether sex-specific gut dysbiosis contributes to these dimorphic brain and immune responses has not been examined. Sex-stratified designs in both preclinical and clinical studies will be essential to avoid missing biologically relevant effects. Second, the developmental memory of perinatal microbial perturbations and its potential impact on lifelong immunity and neurological function remain poorly defined. Clinical and preclinical data are sparse and inconsistent. After caesarean section, overall microbiome structure normalizes as the gut matures although selected genera differ at five years [93]. Rodent prebiotic supplementation from P5 to P14/15 produces a community that is no longer detectable in adulthood [74], whereas perinatal fructo-oligosaccharides in pigs persistently modulate the adult faecal microbiota at nine months [73]. A large population-based study did not find a consistent association between adult microbiome composition and early-life events [94]. The absence of an enduring microbial signature does, however, not exclude a "developmental priming", by which transient perturbations during critical windows shape immune programming with lifelong consequences. This is, for example, illustrated by maternal fiber deprivation, which alters offspring microbiota and predisposes to low-grade inflammation and obesity [95]. HI insults superimposed on antibiotic exposure may therefore unmask or amplify physiologically programmed trajectories that would otherwise remain silent.

Methodologically, preclinical work needs to move beyond broad manipulations such as antibiotic cocktails and germ-free recolonization towards defined microbial communities. Adult rodent studies have already shown that the choice of antibiotic affects outcome [96]. Against this background, a central finding is that neonatal HI-induced *Proteobacteria*-dominant dysbiosis associated with barrier-disruption and LPS-TLR4-pathway driven neuroinflammation can be recapitulated by FMT alone [3], establishing causality. The next step is to define which specific taxa, metabolites and immune-cell intermediates carry this signal and which postnatal time points are amenable to intervention. This will require confirmation of microbial manipulation, attention to maternal–pup transfer, dose calibration, disease-phase-resolved analyses, and long-term behavioural readouts at developmentally appropriate time points.

Two mechanistic links deserve further emphasis. Despite extensive evidence in adult stroke that microbiota-shaped T cells and neutrophils contribute to ischemic brain injury, this axis has not been systematically investigated in neonatal HI. Given the developmental differences between adult and neonatal immune systems, the relevant mechanisms will need to be defined in neonatal models. A related issue is the gut–meningeal axis, which is established only at the beginning of postnatal life and may be particularly vulnerable to disruption by neonatal HI. In addition to these basic questions, clinically common scenarios should be incorporated in future pre-clinical studies. Inflammation-sensitisation of HI brain injury, in which pre- or peri-insult inflammatory hits lower the injury threshold and hypothermia’s therapeutic effects [97, 98]. The additional impact of this condition on gut and microbiome development remains to be investigated. Considering that NEC is increasingly recognized as a disorder of the gut–brain axis affecting neurodevelopment [99], a potential overlap with NEC might be considered as a an additional effect modifier, even though the co-occurrence of NEC and HIE is low.

Clinical studies will be central to overcome limitations of animal models and to capture human genetic and environmental diversity. Systems-biology approaches developed for preterm infants [14, 37] provide a template for multi-omic, longitudinal characterisation of immune trajectories integrating cellular, soluble and microbial dimensions along with behavioural and neurobiological endpoints. Analogous frameworks could be applied to NE cohorts, despite lower incidences and challenges associated with small cohort sizes. An additional challenge is that all HIE/HT infants in published studies received antibiotics whereas controls largely did not, making the effects of HI, hypothermia and antibiotic exposure difficult to disentangle. In the clinical HIE setting, most cooled infants are treated with antibiotics in the suspicion of sepsis, even though the incidence of early-onset sepsis is very low in HIE [100]. Notably, differences exist in antibiotic strategies between different centres [100], which is important to be considered, since antibiotic class and duration are not interchangeable variables and may be central effect modifiers in their own. Future clinical cohorts should record and stratify by antibiotic class and days of exposure rather than treating any-antibiotic exposure as a binary covariate. Comparability between multiple centres is further complicated by NICU-specific microbial communities [101] and by environmental cross-acquisition of probiotics within hospital settings [38]. The PRIMAL trial documented substantial co-bedding-mediated and unit-level cross-exposure [38], raising the still-unresolved question of whether introducing probiotics in the NICU "seeds" the unit and modifies the microbiome of untreated infants. Solutions include matched antibiotic-exposed control groups, multi-center standardization of feeding, sampling protocols and careful documentation of unit-level microbial environments including feeding regimes.

A specific and, to date, largely neglected variable in pre-clinical research is therapeutic hypothermia itself. Body temperature is an established modulator of the gut microbiome, with cold exposure consistently altering community diversity, stability and short-chain fatty acid output across species [102]. In cooled neonates, however, the hypothermia-specific effect cannot be isolated, because cooling is obligatory in clinical care. This immediately touches two unresolved clinical question regarding mild encephalopathy and treatment of late-preterm infants, in whom a neuroprotective benefit is unproven and under ongoing debate [103–106]. If cooling for the first 3 days of life carries a cost for microbiome development with unappreciated downstream gut-brain-immune consequences combined with marginal neurodevelopmental benefits for these infants, current discussions about HT in these infants might be revised. Since this can hardly be dissected in patients, it defines a clear need for animal models to isolate the cooling-specific microbiome effect and to inform the risk–benefit balance of cooling in lower-severity populations. Interdisciplinary research combining basic and clinical research will be central.

## Data Availability

No datasets were generated or analysed during the current study.
